# Chemotherapy-Free Treatment with Radiotherapy and Immunotherapy for Locally Advanced Non-Small Cell Lung Cancer

**DOI:** 10.3390/cancers17091524

**Published:** 2025-04-30

**Authors:** M. Zeeshan Ozair, Balazs Halmos, Angelica D’Aiello, Jaewon Yun, Andrea R. Filippi, Andreas Rimner, Steven H. Lin, Charles B. Simone, Nitin Ohri

**Affiliations:** 1 Montefiore-Einstein Comprehensive Cancer Center, Bronx, NY 10461, USA; bahalmos@montefiore.org (B.H.); adaiell@montefiore.org (A.D.); jaewon.yun@einsteinmed.edu (J.Y.); nitin.ohri@einsteinmed.edu (N.O.); 2 Department of Oncology and Hematology-Oncology, Fondazione IRCCS Istituto Nazionale dei Tumori, University of Milan, 20133 Milano, Italy; andreariccardo.filippi@istitutotumori.mi.it; 3 Department of Radiation Oncology, Medical Center—University of Freiburg, Faculty of Medicine, University of Freiburg, German Cancer Consortium (DKTK), Partner Site DKTK-Freiburg, 79106 Freiburg, Germany; andreas.rimner@uniklinik-freiburg.de; 4 Department of Thoracic Radiation Oncology, The University of Texas MD Anderson Cancer Center, Houston, TX 77030, USA; shlin@mdanderson.org; 5 New York Proton Center, New York, NY 10461, USA; csimone@nyproton.com; 6 Memorial Sloan Kettering Cancer Center, New York, NY 10461, USA

**Keywords:** LA-NSCLC, immunotherapy, chemotherapy-free, radiation therapy

## Abstract

**Background:** Concurrent chemoradiotherapy (CRT) followed by immunotherapy is a standard treatment for locally advanced non-small cell lung cancer (LA-NSCLC), yet many patients are ineligible due to treatment-related toxicity or poor functional status. Chemotherapy-free approaches using radiotherapy (RT) and immunotherapy may offer a safer and equally effective alternative in select patient populations. **Methods:** A comprehensive literature review was conducted using PubMed, Google Scholar, and relevant conference proceedings focusing on trials between 2000 and 2024. Studies investigating chemotherapy-free regimens combining RT and immunotherapy in LA-NSCLC were analyzed, with emphasis on clinical outcomes, biomarker use, treatment sequencing, radiation dose/fractionation, and safety. **Results:** Multiple Phase I/II trials reported promising efficacy with one-year progression-free survival (PFS) ranging from 39% to 76%. Toxicity was generally acceptable, though higher-grade adverse events were more frequent in older, frail populations. Trials integrating PD-L1 expression, tumor mutational burden (TMB), and circulating tumor DNA (ctDNA) showed potential for improved patient stratification. Variation in immunotherapy timing (induction, concurrent, or consolidation) and radiation schedules highlight the need for optimization. **Conclusions:** Chemotherapy-free regimens represent a promising treatment strategy for patients with LA-NSCLC, especially those that are ineligible for standard CRT. Biomarker-driven patient selection and the rational integration of RT and immunotherapy are critical to improving outcomes. Randomized trials are warranted to establish the efficacy and safety of these emerging approaches.

## Simple Summary

This article explores new treatment strategies for patients with advanced non-small cell lung cancer that has not spread. Traditionally, these patients receive a combination of chemotherapy and radiation, but many cannot tolerate chemotherapy due to age or health conditions. We review studies investigating whether radiation combined with therapies which help the immune system fight cancer could be a safer and effective alternative. Early results show that this approach may work well and cause fewer side effects; however, more large-scale studies are needed to confirm these early results.

## 1. Introduction

Immunotherapy has transformed the treatment of lung cancer, including locally advanced non-small cell lung cancer (LA-NSCLC). Following the initial reports from the landmark PACIFIC trial [1], consolidation immunotherapy following chemoradiation (CRT) has become the standard of care for most patients. While the PACIFIC trial demonstrated clear improvements in disease control and overall survival, numerous unmet needs still exist [2]. Treatment with the PACIFIC regimen carries significant toxicity risks, and disease recurrence following treatment remains common. Randomized studies looking to improve disease control by initiating immunotherapy during chemoradiotherapy have been unsuccessful [3,4]. Additionally, approximately 50% of LA-NSCLC patients are unable to receive the PACIFIC regimen due to being unfit for chemoradiotherapy or developing early disease progression or treatment-related toxicity following chemoradiotherapy [5,6]. Some patient subgroups, such as those whose tumors have EGFR mutations or no PD-L1 expression, may derive minimal benefit from consolidation immunotherapy. Here, we review evidence suggesting that chemotherapy-free treatment approaches with radiotherapy and immunotherapy can address some of these issues.

PACIFIC was a randomized, double-blind, placebo-controlled Phase III trial that evaluated the efficacy of immunotherapy with a PD-L1 inhibitor (durvalumab) in patients with unresectable stage III NSCLC that had not progressed after concurrent CRT [1]. Patients were randomized (2:1) to receive either durvalumab or placebo every 2 weeks for up to 12 months as consolidation therapy. A total of 713 patients were enrolled. The median progression-free survival (PFS) duration was 17 months with durvalumab versus 6 months with placebo, and PFS improvement was demonstrated across predefined subgroups based on demographics and disease characteristics. This led to the approval of durvalumab by regulatory authorities as a standard treatment option in this setting. Updated analyses confirm the durable overall survival (OS) and PFS benefit in patients receiving durvalumab, with an estimated 43% of patients assigned to durvalumab remaining alive at 5 years, compared to 33% of the placebo group [2]. Several studies since PACIFIC, including GEMSTONE-301 (a randomized trial testing a PD-1 inhibitor versus placebo) [7], support the administration of consolidation immunotherapy following CRT.

Based on the concerns listed above and landmark studies demonstrating that immunotherapy can be more effective than chemotherapy in advanced NSCLC (e.g., KEYNOTE-024 [8] and Checkmate227 [9]), there is growing interest in chemotherapy-free approaches utilizing radiotherapy and immunotherapy for the treatment of LA-NSCLC. While outcomes in initial studies have generally been favorable, the optimal design of a chemotherapy-free treatment regimen for LA-NSCLC remains unknown. Broad considerations include the selection of immunotherapeutic agents, timing of radiotherapy relative to immunotherapy, and radiotherapy parameters, including treatment technique and dosing. Special considerations come into play when combining radiotherapy and immunotherapy, as the two modalities may act synergistically, but there is also evidence that radiotherapy may attenuate the effects of immunotherapy in some settings.

To compile a comprehensive list of articles on this topic, a systematic search was conducted using PubMed and Google Scholar. Search terms included “LA-NSCLC”, “chemotherapy-free treatment”, “unresectable”, “immunotherapy”, “checkpoint inhibitors”, “radiation”, “radiotherapy”, and “proton therapy”. Studies published between 2000 and 2024 were considered. Additionally, references from key publications and abstracts from relevant conferences, including the European Society of Medical Oncology (ESMO Open and *Annals of Oncology*), were reviewed and incorporated where appropriate. Public trial registries were reviewed for the status of ongoing Phase I and II trials.

## 2. Mechanism of Synergy Between Immunotherapy and Radiation

**Figure 1:**
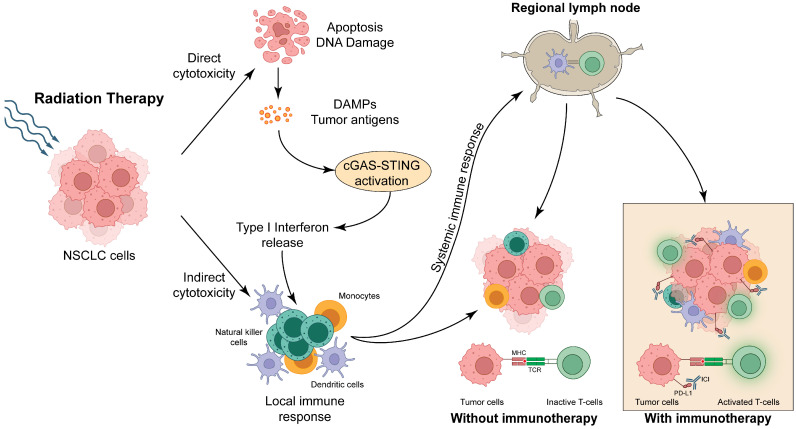
Direct and indirect cytotoxic effects induced by RT. Immunotherapy when used in a specific sequence with RT induces optimal activation of innate and humoral immunity, which promotes tumor cell death both at the primary tumor site and any secondary sites (abscopal effect). PD-L1 immunotherapy is shown as an example; however, other immune checkpoint inhibitors have a similar mechanism of action.

Radiation therapy (RT) is known to exert anti-tumor effects via both direct and indirect cytotoxic mechanisms (Figure 1). RT can induce apoptosis of tumor cells in the target field by damaging the genome of tumor cells, inducing a G1/S block and activation of cell intrinsic and extrinsic proapoptotic pathways [10]. In addition, RT can also induce local and systemic immune responses by promoting the release of tumor antigens and danger-associated molecular patterns (DAMPs) [10,11]. The process involves the expulsion of nuclear and mitochondrial DNA into the cytosol and activation of the cyclic GMP-AMP synthase (cGAS)-stimulator of interferon genes (STING) pathway. The cGAS-STING cascade leads to the activation of Type I interferons, which activate dendritic cells and promote T-cell responses against the tumor, in turn enhancing both innate and humoral immune responses (Figure 1). This systemic response induced by RT can lead to enhanced infiltration of tumor-infiltrating lymphocytes outside the target field, known as the abscopal effect. Immune checkpoint inhibitors, such as those that inhibit PD-1 and PD-L1 interactions or CTLA4 and CD80/86 interactions, can synergistically enhance the effectiveness of RT by promoting local and systemic immune responses [10,12].

Together, these findings support the possibility of synergy between RT and immunotherapy in the treatment of LA-NSCLC. Randomized trials aiming to demonstrate that this synergy can impact clinical outcomes have had mixed results [13,14], which may be related to the differences in patient populations (e.g., prior immunotherapy exposure) and radiotherapy schedules. Of note, both the PACIFIC trial [1] and the ADRIATIC trial (for limited stage small cell lung carcinoma) [15] demonstrated benefits when immunotherapy was initiated after chemoradiation, suggesting that a sequential timing of RT and immunotherapy may be optimal.

## 3. Chemotherapy-Free Treatment Regimens for Advanced/Metastatic NSCLC

The notion of pursuing chemotherapy-free regimens in advanced/metastatic NSCLC is now supported by multiple trials, including KEYNOTE-024 [8]. KEYNOTE-024 was an open label Phase III clinical trial that investigated the efficacy of pembrolizumab compared to platinum-based chemotherapy in patients with metastatic NSCLC with PD-L1 TPS of 50% or greater. The study enrolled 305 participants and demonstrated that pembrolizumab significantly improved both PFS (median 10 months v. 6 months) and OS (five-year OS 32% v. 16%) compared to chemotherapy [16]. Additionally, participants treated with immunotherapy had fewer severe adverse events and improved quality-of-life outcomes. These results support pembrolizumab monotherapy as an effective first-line treatment option in patients with metastatic NSCLC with high PD-L1 expression.

CheckMate 227 evaluated the efficacy of a combination of nivolumab and ipilimumab for first-line treatment of patients with advanced NSCLC [9]. The trial demonstrated that the combination of nivolumab and ipilimumab significantly improved overall survival compared to standard platinum-doublet chemotherapy in patients with metastatic NSCLC, most notably in those with a high tumor mutational burden (TMB) and PD-L1 expression ≥1%. Based on these results, the FDA approved the combination of nivolumab and ipilimumab for the first-line treatment of metastatic NSCLC patients with PD-L1 expression of ≥1%.

These trials demonstrate that immunotherapy can be more effective than chemotherapy for biomarker-selected NSCLC patients. While chemotherapy and immunotherapy are often used together in the first-line setting for advanced NSCLC, many patients are now treated with a chemotherapy-free approach when the risk/benefit ratio of adding chemotherapy is deemed to be unfavorable. Similar considerations could apply to the setting of LA-NSCLC.

## 4. Completed Clinical Trials Exploring Chemotherapy-Free Regimens in LA-NSCLC

**Figure 2:**
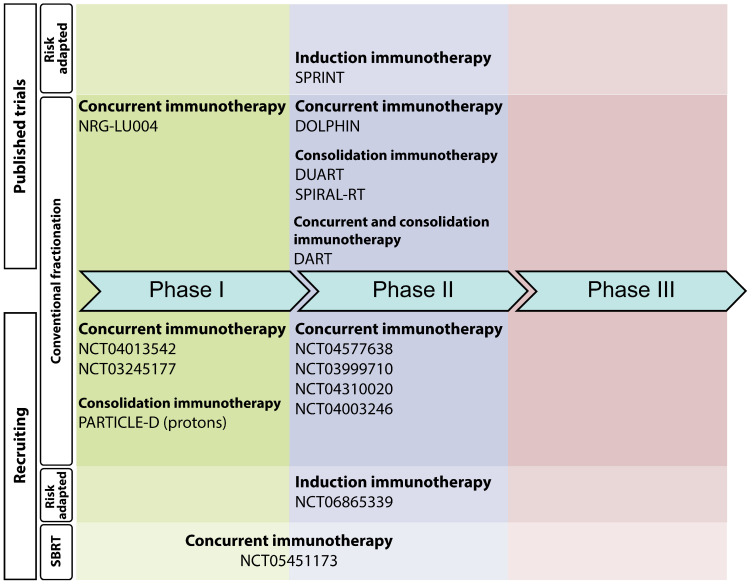
Ongoing and published trials of chemotherapy-free regimens for LA-NSCLC.

Several recently completed Phase I/II trials have explored the utility of immunotherapy in combination with radiotherapy and without chemotherapy as curative-intent treatment for LA-NSCLC. These studies are summarized in the text below, as well as in Table 1 and Figure 2.

SPIRAL-RT was a Japanese trial that evaluated the safety and efficacy of radiotherapy followed by consolidation durvalumab in patients with LA-NSCLC with PD-L1 expression of ≥25% [17,18]. It was a single-arm trial where 33 participants received a radiation dose of 54–66 Gy, followed by durvalumab (10 mg/kg every 2 weeks for up to 12 months). Key outcomes included a one-year PFS rate of 39% and a one-year OS rate of 72%, with some suggestion that non-squamous histology and high PD-L1 expression were favorable prognostic factors. Two participants had Grade 4 or 5 toxicities that led to discontinuation of treatment, and the most common adverse events observed overall were radiation pneumonitis and lung infections.

SPRINT was another trial where radiotherapy and immunotherapy were employed to treat biomarker-selected patients with LA-NSCLC [19]. Study participants with PD-L1 expression of ≥50% were treated with three cycles of induction pembrolizumab, followed by accelerated and risk-adapted radiotherapy, followed by consolidation pembrolizumab for up to 13 cycles. Risk-adapted radiotherapy was implemented as follows. Based on PET/CT performed after induction immunotherapy, tumors and lymph nodes with metabolic volumes exceeding 20 cc were treated with a standard dose of 55 Gy in 20 fractions, while smaller targets received a lower dose of 48 Gy in 20 fractions. The one-year PFS rate was 76%, and the one-year OS rate was 92%. No Grade 4 or 5 treatment-related adverse events (AEs) were reported, and Grade 3 treatment-related AEs were infrequent.

DOLPHIN was a Japanese trial with less stringent inclusion criteria with respect to PD-L1 expression [20]. Thirty-five participants with PD-L1 TPS of 1% or higher were treated with conventional radiotherapy (60 Gy in 30 fractions) in combination with concurrent and consolidation durvalumab (10 mg/kg every 2 weeks) for up to one year. The one-year PFS rate was 72%, and the one-year OS rate was 94%. Two Grade 5 adverse events were reported (6%).

The studies summarized above tested chemotherapy-free treatment in biomarker-selected patients who would generally have been eligible for standard chemoradiotherapy. Additional studies, summarized below, have included patients deemed to be unfit for chemoradiotherapy, without selection based on PD-L1 expression.

DUART assessed the safety and tolerability of durvalumab following radiotherapy in patients with unresectable LA-NSCLC who were deemed ineligible for chemotherapy [21,22]. DUART included a cohort of patients who were treated with palliative radiotherapy doses (<54 Gy) and a cohort of patients who were treated with definitive radiotherapy (54 to 66 Gy). Both cohorts subsequently received durvalumab (1500 mg every 4 weeks) for up to 12 months. In the cohort of patients treated with definitive radiotherapy, the one-year PFS rate was 47%, and the one-year OS rate was 64% [22]. Trends suggesting better outcomes in patients with PD-L1-positive disease were observed [23]. Moreover, exploratory circulating tumor DNA (ctDNA; see below) analyses showed that ctDNA clearance during maintenance immunotherapy was associated with favorable clinical outcomes [24].

DART also evaluated treatment with radiotherapy and durvalumab in patients with LA-NSCLC who were thought to be ineligible for standard chemoradiotherapy [24]. Unlike in DUART, durvalumab was started concurrently with radiotherapy. The one-year PFS rate was 75%, and the one-year OS rate was 42% [25]. In an interim analysis, Grade 3 or higher adverse events were reported in 3 out of 27 participants, all of whom had pneumonitis [26].

**Table 1:** Completed trials testing chemotherapy-free treatment with radiotherapy and immunotherapy for LA-NSCLC.

Registration #, Trial Name	Sample Size	Phase	Primary Endpoint	Immunotherapy Schedule and Timing	RT Schedule	Patient/Biomarker Selection	Key Findings
jRCT2080224763 DOLPHIN [20]	35	II	1-year PFS rate	Durvalumab, every two weeks for one year, starting concurrent with RT	60 Gy in 30 fractions	PD-L1 TPS ≥ 1%	1-year PFS = 72% 1-year OS = 94%
NCT04249362 DUART [21,22] (Cohort A)	53	II	Safety	Durvalumab, every four weeks for one year, starting after RT	60 Gy (+/−10%)	Ineligible for chemotherapy	7 Grade 3–5 adverse events related to study therapy 1-year PFS = 47% 1-year OS = 64%
JMA-IIA00434 (jRCT) SPIRAL-RT [17,18]	33	II	1-year PFS rate	Durvalumab, every two weeks for one year, starting after RT	54 to 66 Gy in 27 to 33 fractions	Ineligible for concurrent chemo-radiotherapy	1-year PFS = 39% 1-year OS = 72%
NCT03523702 SPRINT [19]	25	II	1-year PFS rate	Pembrolizumab, every three weeks, before RT (3 cycles) and after RT (12 cycles)	48 or 55 Gy in 20 fractions (risk-adapted)	PD-L1 TPS ≥ 50%	1-year PFS = 76% 1-year OS = 92%
NCT03999710 DART [26]	27	II	2-year PFS rate	Durvalumab, every four weeks for one year, starting concurrent with RT	54–66 Gy in 27–33 fractions	Ineligible for concurrent chemo-radiotherapy	1-year PFS = 42% 1-year OS = 75%
NCT04003246 [27]	10	II	PFS rate	Durvalumab, every four weeks for one year, starting concurrently with RT.	54–66 Gy in 27–33 fractions	Medically inoperable disease or unwilling to undergo surgery PD-L1 TPS (any)	1-year PFS = 20%
NCT03801902 NRG-LU004 [28]	24	I	Safety	Durvalumab, every four weeks for one year, starting 0–2 weeks before RT start	Cohort 1: 60 Gy in 30 fractions Cohort 2: 60 Gy in 15 fractions	PD-L1 TPS ≥ 50%	No DLTs related to study therapy

The UT Southwestern group explored chemotherapy-free treatment with radiotherapy and durvalumab in patients with LA-NSCLC who were unselected with respect to PD-L1 expression and generally would have been fit for chemoradiotherapy [27]. Participants received a standard radiotherapy dose of 60 Gy in 30 fractions, with durvalumab (1500 mg every four weeks) started at the time of radiotherapy initiation. Results were disappointing, and the study was terminated after ten out of fifty planned subjects were enrolled. One case of Grade 4 renal toxicity was observed, and two participants experienced Grade 5 pulmonary toxicity. The one-year PFS rate was 20%, and the one-year OS rate was 50%.

For one completed trial testing radiotherapy and immunotherapy without chemotherapy for patients with LA-NSCLC with PD-L1 TPS ≥ 50%, efficacy outcomes have not yet been reported. NRG-LU004 (also known as ARCHON-1) was a Phase I clinical trial investigating the combination of durvalumab with either accelerated and hypofractionated radiotherapy (60 Gy in 15 fractions) or conventionally fractionated radiotherapy (60 Gy in 30 fractions) [28]. Twelve participants were enrolled in each cohort, and radiotherapy was initiated within two weeks after the first durvalumab infusion. While study results have not yet been published, investigators have reported that treatment on both arms has appeared to be safe.

The heterogeneous designs of the studies summarized limit our ability to make broad conclusions about treatment efficacy. Not surprisingly, the most favorable clinical outcomes have been observed in trials with biomarker-selected patients without specific contraindications to standard therapy. A randomized study comparing chemotherapy-free treatment with radiotherapy and immunotherapy versus the PACIFIC regimen could be appropriate at this point. However, the optimal radiotherapy and immunotherapy schedules cannot clearly be identified based on available data. For patients who are ineligible for chemoradiotherapy, the clinical outcomes summarized above appear to surpass those seen in recent studies with radiotherapy alone [29]. A randomized trial testing radiotherapy and immunotherapy in frail patients should therefore also be considered.

## 5. Ongoing Chemotherapy-Free Clinical Trials in LA-NSCLC Patients

A review of public trial registries demonstrated that additional Phase I and II trials testing chemotherapy-free treatment of LA-NSCLC utilizing radiotherapy and immunotherapy are ongoing (Table 2 and Figure 2). While the study designs are generally similar to the designs of the completed studies summarized above, some novel elements include the utilization of dual-agent immunotherapy with nivolumab and ipilimumab in one trial (NCT04013542) and treatment with proton beam radiotherapy in another trial (NCT03818776).

To our knowledge, there are no active or completed randomized studies testing chemotherapy-free treatment with radiotherapy and immunotherapy for patients with LA-NSCLC. There are now ample data to support such a trial, and we have witnessed first-hand that most lung cancer patients are excited by the prospect of chemotherapy-free treatment. Considerations for the design of a Phase III trial in this space are described in Section 6.

## 6. Considerations for Future Studies of Chemotherapy-Free Treatment of LA-NSCLC

**Figure 3:**
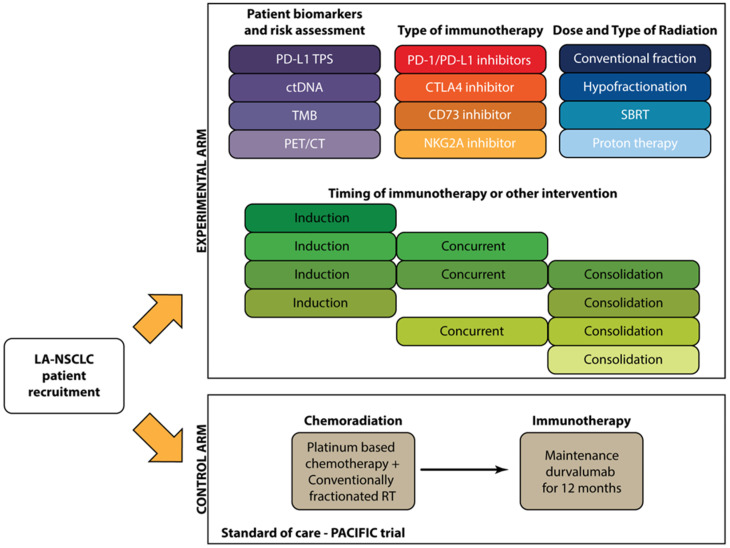
Considerations in design of Phase III trials for chemotherapy-free regimens for LA-NSCLC.

Here, we summarize some key considerations for future studies testing chemotherapy-free treatment of LA-NSCLC with radiotherapy and immunotherapy (summarized in Figure 3). Of note, we do not believe that these and other studies utilizing definitive radiotherapy should be restricted to patients with “unresectable” disease, as randomized trials have not demonstrated that surgical resection improves outcomes in LA-NSCLC [30]. This is certainly a controversial topic, and we support multidisciplinary evaluation for all patients with LA-NSCLC.

### 6.1. Patient Selection

Two categories of LA-NSCLC patients could be most suitable for chemotherapy-free treatment:

(1) Patients who are deemed to be ineligible for standard chemoradiotherapy

Establishing criteria for chemoradiotherapy ineligibility poses a significant challenge. Factors that may be considered include comorbid medical conditions, performance status, and thoracic disease burden. In our experience, many relatively frail patients can tolerate low-dose weekly carboplatin and paclitaxel during thoracic radiotherapy. Tools that identify patients who are unlikely to tolerate high-dose cisplatin [31] therefore have limited utility in this setting. Nonetheless, many patients with LA-NSCLC are deemed to be ineligible for chemotherapy or refuse chemotherapy and treated with thoracic radiotherapy alone. Radiotherapy intensification does not seem to improve outcomes in this setting [29], so new systemic therapy options for this patient population are sorely needed.

(2) Patients for whom chemotherapy-free treatment is expected to yield superior outcomes compared to standard chemoradiotherapy.

The omission of chemotherapy for patients expected to respond well to immunotherapy might reduce treatment toxicity without affecting disease control. While we currently have few reliable predictors of immunotherapy efficacy (See Section 6.2), emerging biomarker research may help better define this patient population. Treatment sequencing may also affect outcomes, as two randomized trials for patients with melanoma have demonstrated that immunotherapy may be most effective when administered before local therapy [32,33].

### 6.2. Identification of LA-NSCLC Patients Most Likely to Benefit from Immunotherapy

Current guidelines for first-line treatment with immunotherapy and without chemotherapy in the metastatic NSCLC setting are based on PD-L1 expression, with patients with a high PD-L1 expression (i.e., TPS > 50%) often being recommended to receive immunotherapy without chemotherapy [34]. Similar considerations may apply in the design of LA-NSCLC trials involving immunotherapy [35]. Indeed, PACIFIC demonstrated differential benefits of adjuvant immunotherapy following chemoradiotherapy depending on PD-L1 expression. Subgroup analyses of PACIFIC revealed that patients with PD-L1 TPS ≥ 1% had greater benefit from durvalumab after CRT (HR for OS of 0.61) compared to those with PD-L1 TPS < 1% or unknown status (HR for OS 1.15) [1]. The benefit of durvalumab was even more pronounced in patients with PD-L1 TPS ≥ 25% [2]. However, PD-L1 expression can be affected by variability in tissue sampling, antigen detection, and sampling errors, and thus by itself is not an ideal biomarker [35]. Due to these limitations, alternative methods of quantifying PD-L1 expression are also being explored, such as blood exosomal levels, ELISA, and gene expression levels in the tissue. In addition, there are several emerging biomarkers beyond PD-L1 that are being investigated to predict immunotherapy response in patients some of which, such as TMB, MSI, and ctDNA are discussed below. It is worth noting that a combination of these assays may ultimately be a better predictor of immunotherapy response than either in isolation.

TMB quantifies the amount of somatic mutations in the entire tumor genome and is determined by next-generation sequencing of tumor samples. It has been investigated as a predictive biomarker for immunotherapy efficacy in mainly the metastatic setting. A high TMB of ≥ 10 mutations/megabase (mt/MB) has been consistently associated with improved clinical outcomes for patients receiving immunotherapy in this cohort, as observed in CheckMate 227 and several retrospective studies [36,37,38]. There is emerging interest in evaluating the role of TMBs in the setting of non-metastatic LA-NSCLC as well. In a recent retrospective study, 81 patients with stage III NSCLC treated with CRT and durvalumab were grouped into either TMB-high (≥10 mt/Mb) or TMB-low (<10 mt/Mb) groups [39]. Patients with TMB-high tumors were found to have significantly lower 24-month locoregional failure (LRF) following treatment (9% vs. 51%) as well as improved 24-month PFS (66% vs. 27%) when compared to TMB-low patients. Thus, high TMB may complement PD-L1 expression as a biomarker to identify patients most likely to respond to immunotherapy, and further prospective trials are needed to validate its predictive value in the context of LA-NSCLC.

Studies have also shown that mismatch repair deficiency (dMMR) and microsatellite instability (MSI) can also be predictive of response to immunotherapy agents [40,41]. However, both MSI and TMB are not currently widely used in clinical trial designs because they share several limitations when it comes to tissue availability for detection, clinical interpretation, and differences depending on the stage of disease the tissue is being sampled. Moreover, MSI is fairly uncommon in the LA-NSCLC setting.

Another tumor biomarker that is currently under investigation is circulating tumor DNA (ctDNA) [42]. ctDNA has emerged as a promising biomarker for detecting treatment response, minimal residual disease (MRD), and recurrence in locally advanced non-small cell lung cancer (LA-NSCLC). Studies have shown the utility of ctDNA dynamics during treatment and post-radiation phases, particularly for identifying patients who may benefit from therapy escalation or those at risk for disease recurrence [43]. Changes in ctDNA levels during treatment are linked to clinical outcomes in LA-NSCLC and ctDNA positivity following radiation is associated with a markedly higher risk of recurrence and poorer survival outcomes [44]. Moreover, ctDNA response during immunotherapy has shown to identify patients with LA-NSCLC who are likely to respond to treatment. Patients with negative ctDNA one month after CRT show better PFS and OS compared to those with positive ctDNA detection [45]. In the neoadjuvant setting, ctDNA clearance during therapy is associated with improved clinical outcomes as shown in the CheckMate-816 trial [46]. The trial evaluated neoadjuvant nivolumab plus chemotherapy in patients with resectable NSCLC. Patients who achieved ctDNA clearance exhibited prolonged event-free survival (EFS) compared to those with persistent ctDNA. This highlights ctDNA clearance as a potential marker of therapy response and a predictor of improved prognosis. In contrast, the absence of ctDNA clearance correlates with a higher risk of disease progression or relapse, emphasizing its clinical relevance in treatment decision-making during neoadjuvant therapy. Moreover, post-definitive RT, MRD detection through ctDNA testing has been identified as a significant prognostic factor for progression-free survival [43]. Importantly, ctDNA dynamics provide an earlier indication of disease recurrence compared to imaging-based assessments, with lead times up to 5.5 months [43,47]. Serial ctDNA monitoring may thus allow clinicians to intervene earlier, potentially modifying treatment strategies to improve patient outcomes.

Thus, ctDNA dynamics may be a useful biomarker in future trials to select patients that are likely to respond to immunotherapy, to quantify treatment response, and to individualize treatment plans that omit chemotherapy.

### 6.3. Radiation Type and Dose

RTOG 0617 demonstrated that for patients with LA-NSCLC receiving concurrent chemoradiotherapy, radiotherapy dose escalation from 60 to 74 Gy led to decreased survival rates [48]. A secondary analysis of RTOG 0617 implicated the extent of irradiation of the “immune system” as a reason for inferior outcomes on the high-dose arm [49]. Of note, RTOG 0617 utilized conventional radiotherapy fractionation, such that dose escalation involved prolongation of the treatment course. Two retrospective studies have shown that hypofractionated RT in the context of concurrent chemotherapy is associated with reduced risk of severe lymphopenia and improved clinical outcomes patients with LA-NSCLC [50,51]. In a recent randomized study, hypofractionation along with risk-adapted radiotherapy dose reduction reduced the rate of Grade 3–4 lymphopenia from 81% to 48% [52].

The trials discussed above utilized slightly different treatment regimens of conventionally fractionated RT. Most trials employed conventionally fractionated radiotherapy, though some, such as SPRINT and a cohort from NRG-LU004, utilized hypofractionation. Although available evidence supports the use of hypofractionated RT in the context of immunotherapy, hypofractionation along with dose escalation may have excessive toxicity risks. While large, randomized trials will be needed to identify the optimal radiotherapy regimen to use in the context of immunotherapy and without chemotherapy for LA-NSCLC, we believe that existing evidence supports the use of relatively short radiotherapy courses. An additional consideration for future trials is the type of radiation employed. A meta-analysis of randomized studies from the Meta-Analysis of Radiotherapy in Lung Cancer (MAR-LC) collaborative group indicated that accelerated fractionated RT was linked with enhanced long-term survival (hazard ratio = 0.88; *p* = 0.009), resulting in an absolute benefit of 2.5% (8.3% to 10.8%) at 5 years [53]. Although patients from all stages of lung cancer were included in this meta-analysis, about 80% of the patients analyzed had LA-NSCLC. This is in contrast to some Phase III trials that showed no benefits of accelerated fractionated RT either in the context of hyperfractionation (such as CHARTWEL and ECOG 2597) [54,55] or moderate hypofractionation [56,57] for conventional chemoradiation regimens. Moreover, RTOG 0617 showed that dose escalation beyond 60 Gy demonstrated worse survival outcomes for the high dose arms compared to standard-dose arms [48]. New trials will likely be required to assess the benefits of altered fractionation regimens in the context of immunotherapy. The NRG LU004 trial described above has demonstrated that both accelerated and conventionally fractionated RT are safe when combined with durvalumab in patients exhibiting PD-L1 expression > 50% [28]. The trials discussed above have employed both conventionally fractionated and accelerated hypofractionated regimens as part of their chemotherapy-free regimens. Ongoing clinical trials are exploring the possibility of hypofractionated SBRT regimens (Table 2). In this context, NRG-LU008 is an ongoing Phase III randomized clinical trial that is exploring treating the primary tumor with SBRT followed by concurrent chemoradiation to the mediastinum and consolidation immunotherapy [58].

Proton therapy has previously been shown to have a reduced cardiac dose relative to intensity-modulated radiotherapy (IMRT) [59]. Hence it raises the question of whether proton radiation could be used in chemotherapy-free regimens to further reduce toxicity. Further clinical trials are currently ongoing to evaluate the safety and potential survival benefits between proton RT and photon RT. The PARTICLE-D (NCT03818776) trial is assessing the safety of treating patients with the combination of accelerated proton beam therapy and durvalumab. Patients will either receive 60 Gy equivalent in 20 fractions or 69 Gy equivalent in 23 fractions, followed by durvalumab monotherapy, and they will be assessed for dose limiting toxicities (DLTs).

### 6.4. Safety and Toxicity

For the reasons mentioned in the previous section, the dose and fractionation of RT used in future clinical trials are important considerations in the context of LA-NSCLC for efficacy considerations and patient safety and toxicity concerns. Data from NRG-LU004 have been generally encouraging with regards to accelerated hypofractionated RT. As briefly discussed above, in the accelerated fractionated RT cohort, 4 patients developed Grade 3 AEs, one Grade 4 (lymphopenia), and one Grade 5 AE (pneumonia unrelated to treatment) compared to conventional RT, where there were 8 Grade 3 AEs and 1 Grade 5 AE (respiratory failure unrelated to treatment).

In the context of chemotherapy-free regimen using conventional fractionation, existing trials have all shown limited toxicity from combining RT with immunotherapy. For example, in SPIRAL-RT only two of the 33 patients had Grade 4 (neutropenia) or Grade 5 (pneumonia) toxicities. Although the most common AE of any grade in this study was radiation pneumonitis, that was likely related to the fact that the patient recruited for the trial were older (median age of 79 years) and had a poorer performance status that made them ineligible for chemotherapy. The DOLPHIN trial reported radiation pneumonitis related Grade 3 or 4 toxicities of 11.8% which were higher than that reported in the PACIFIC (3.4%) or KEYNOTE-799 trials (8.0%), even though Grade 5 AEs were infrequent in this trial (5.9%). The authors suggested that the higher toxicity was likely related to older patient population (median age 72 years), the design of the trial including patients with Grade 2 or higher pneumonitis after RT, and perhaps the incidence of pneumonitis being higher among the Japanese patient cohort. Similarly, in the DUART trial, which had a median age of recruitment of 79 years, had Grade 3/4 AEs of 9.8% with slightly higher incidence in standard doses compared to palliative doses (11.9% versus 7.0%). Grade 5 PRAEs (adverse events defined as potentially treatment-related) in this trial occurred in 1% of patients (1/102).

In comparison, the SPRINT trial, where the median age of recruited patients was 71 years, had no Grade 4 or 5 AEs, while Grade 3 AEs were infrequent. The DART trial, which had a higher median age of recruitment of 80.6 years, had only 3/27 patients developed serious AEs that were treatment-related (1 patient with Grade 3 pneumonitis, 1 patient with Grade 3 pneumonia and pulmonary embolism, and 1 patient with Grade 5 pneumonitis) [26]. Notably, the recently published NCT04003246 [27] was closed early due to high incidence of poor clinical outcomes when durvalumab was initiated concurrently with radiation therapy, with 1 patient experiencing a Grade 4 AE of acute kidney injury and 5 patients experiencing Grade 3 pulmonary toxicities. Moreover, 5 patients had disease progression at a median of 6 months while 2 patients died of pulmonary toxicity. The median age of patients in this trial was 65.5 years. The negative results from this trial reinforce the idea that optimal benefit from immunotherapy may be derived from sequential timing relative to RT rather than concurrent timing.

Several clinical trials listed in Table 2 continue to assess the safety and toxicity of various RT regimens in combination with immunotherapy. Our current opinion is that thoracic radiotherapy and immunotherapy have established toxicity risks, and there is little evidence that those risks increase more than in an additive fashion when radiotherapy and immunotherapy are used concurrently. Key variables that affect toxicity risks for a specific patient, which include thoracic disease burden, radiotherapy plan parameters, and medical comorbidities, must be analyzed carefully before more specific conclusions can be drawn.

### 6.5. Type and Order of Immunotherapy

Immunotherapy has shown potential as a radiosensitizer in cancer treatment, enhancing the effects of radiation therapy through immune system activation and synergistic mechanisms [12]. However, as discussed above, ionizing radiation can potentially suppress the local immune response by eliminating lymphocytes, which are highly sensitive to radiation, from the treatment field. Indeed, patients undergoing RT often experience treatment-related lymphopenia. Preclinical studies have demonstrated that cytotoxic T-cells are more sensitive to RT compared to regulatory T-cells [12,50,60]. This can result in a higher proportion of regulatory T-cells, which suppresses the immune response [61]. This might explain the high incidence of disease progression observed when immunotherapy is given concurrently with radiation therapy in some studies discussed above [27]. A clinically pertinent question is whether induction immunotherapy in a chemotherapy-free regimen is as safe and effective as concurrent or sequential regimens following RT. The studies discussed so far have used different combinations of induction, concurrent, and consolidation immunotherapy. We suspect that induction immunotherapy may be particularly useful, due to the considerations above as well as the opportunity to elicit radiographic responses that reduce radiotherapy target volumes. Future studies using this approach are warranted.

Most trials to date have used PD1 and PD-L1 inhibitors such as pembrolizumab, durvalumab, or atezolizumab monotherapy as part of their chemotherapy-free regimen. Adding other classes of immunotherapeutic agents, such as those targeting CTLA-4 and LAG-3, may further improve treatment efficacy. As an example, in advanced melanomas, targeting LAG-3 has demonstrated synergistic effects in immunotherapy combinations, leading to enhanced T-cell activation and improved outcomes [62]. Dual immune checkpoint inhibition may amplify immune responses, and ongoing research may extend these benefits to LA-NSCLC, particularly in biomarker-selected populations. With the availability of novel inhibitors including an anti-CD73 monoclonal antibody (oleclumab) and the anti-NKG2A monoclonal antibody (monalizumab), recent studies have addressed whether combinations of immunotherapy have an additive benefit over monotherapy. The COAST trial was a Phase II trial designed to assess the efficacy and safety of adding immunotherapy agents to standard treatment for patients with unresectable Stage III NSCLC who have not progressed following CRT [63]. Participants were randomized into one of three treatment arms: (1) durvalumab alone (standard of care), (2) durvalumab + monalizumab and, (3) durvalumab + oleclumab. While durvalumab alone group demonstrated a median PFS of 6.3 months, the durvalumab/monalizumab group showed a median PFS of 15.1 months and the durvalumab/oleclumab group had a median PFS of 11.5 months. Both dual immunotherapy groups showed a significant improvement over the durvalumab alone group, with no significant increases in toxicity.

Although the COAST trial utilized CRT followed by immunotherapy in its study design, the results are supportive of future trials with chemotherapy-free regimen utilizing dual agent immunotherapy with durvalumab/monalizumab or durvalumab/oleclumab. A related Phase II study, NeoCOAST [64] investigated the role of neoadjuvant (pre-surgical) therapy with durvalumab, durvalumab/monalizumab, or durvalumab/oleclumab for patients with resectable early-stage NSCLC. While durvalumab monotherapy achieved an MPR of approximately 11%, combining durvalumab with either oleclumab or monalizumab significantly improved MPR rates to 19–30%. These combinations also showed higher rates of pathological complete responses (pCR), indicating greater tumor regression at the time of surgery. The findings suggest that adding novel immunotherapies to anti-PD-L1 therapy can enhance immune response and efficacy in early-stage NSCLC. In contrast, a recent Phase I/II trial in 19 patients with LA-NSCLC treated with concurrent ipilimumab with conventional CRT followed by maintenance nivolumab had to be discontinued due to severe pulmonary toxicity in a majority of the patient cohort [65]. Together, these results suggest that use of CTLA4 inhibitors in the management of LA-NSCLC will need to be designed with toxicity risk mitigation strategies. Several ongoing trials (such as NCT04013542, NCT04929041, and NCT04929041) are exploring the role of ipilimumab either as monotherapy or in combination with PD1/PD-L1 inhibitors in chemotherapy-free regimens for LA-NSCLC (Table 2).

## 7. Future Directions

Moving forward, prospective randomized trials are essential to establish chemotherapy-free regimens as a standard treatment option for patients with locally advanced NSCLC. Such trials should incorporate biomarker-driven selection strategies, including PD-L1 expression, TMB, and ctDNA dynamics, to identify patients most likely to benefit from immunotherapy without chemotherapy. Further studies are also warranted to evaluate the optimal sequencing of immunotherapy relative to radiotherapy, with particular interest in induction strategies that may reduce target volumes and enhance immune priming. The integration of novel immunotherapeutic agents, including dual immune checkpoint blockade and emerging targets such as CD73 and NKG2A, may offer additional clinical benefit, particularly in biomarker-selected populations. In parallel, trials examining the role of altered radiation fractionation and modality—such as hypofractionation and proton therapy—are needed to clarify their impact on immune responses and treatment-related toxicities. Together, these efforts will inform the development of safe, effective, and personalized chemotherapy-free regimens for patients with LA-NSCLC.

## 8. Conclusions

For patients with locally advanced non-small cell lung cancer (LA-NSCLC), chemotherapy-free treatment with radiotherapy and immunotherapy is an emerging treatment approach. There is little evidence that thoracic radiotherapy and immunotherapy carry synergistic toxicity risks. Clinical outcomes in completed studies have generally been favorable, especially in trials that enrolled relatively fit patients and employed biomarker selection. Sequential treatment with immunotherapy and hypofractionated radiotherapy may be the preferred approach to test in a pivotal Phase III trial.

## Figures and Tables

**Table 2:** Ongoing trials testing chemotherapy-free regimens in LA-NSCLC.

Trial Name	Phase	Primary Endpoint(s)	Immunotherapy Schedule and Timing	RT Schedule	Patient/Biomarker Selection	Status
NCT04013542	I	Safety and feasibility	Nivolumab, every three weeks for up to 8 cycles, and ipilimumab every six weeks for up to 4 cycles, both starting with RT.	60 Gy in 30 fractions	Ineligible for concurrent chemoradiotherapy	Active, not recruiting
NCT03818776 (PARTICLE-D)	I	Safety	Durvalumab, every four weeks for one year, starting one week before RT	Arm 1: 60–69 CGyE in 30 fractions (proton radiotherapy)	Ineligible for concurrent chemoradiotherapy	Terminated
NCT05451173	I/II	Safety, PFS	Durvalumab, every four weeks for one year, starting concurrently with RT.	3.5–4.0 Gy × 15 fractions	PD-L1 TPS ≥ 1% (“preferred”)	Not yet recruiting
NCT04577638(AIRING)	II	DCR *	Nivolumab, every two weeks for six months, starting concurrently with RT.	66 Gy in 24 fractions	≥1 of several “fragility criteria”, which include ECOG performance status 2 and age > 74	Recruiting
NCT04310020(SWOG S1933)	II	PFS	Atezolizumab, every three weeks for one year, starting after RT	60 Gy in 15 fractions	Ineligible for concurrent chemoradiotherapy	Recruiting
NCT04351256(Trade-HYPO)	II	Safety and feasibility	Durvalumab, every four weeks for one year, starting concurrently with RT.	55 Gy in 20 fractions	Ineligible for concurrent chemoradiotherapy	Recruitiing
NCT06865339	II	ORR ^†^ to induction immuno-therapy	Cemiplimab and Fianlimab, every three weeks, before RT (3 cycles) and after RT (13 cycles)	Risk-adapted conventional fractionation	Low PD-L1 TPS (<50%)	Recruiting

* DCR: disease control rate, ^†^ ORR: objective response rate.

## Data Availability

Not applicable.

## Abbreviations

AEs: Adverse Events; ALK: Anaplastic Lymphoma Kinase; CD: Cluster of Differentiation; cGAS-STING: Cyclic GMP-AMP Synthase–Stimulator of Interferon Genes pathway; CRT: Chemoradiotherapy; ctDNA: Circulating Tumor DNA; CTLA-4: Cytotoxic T-Lymphocyte Antigen 4; DCR: Disease Control Rate; DLT: Dose Limiting Toxicity; dMMR: Mismatch Repair Deficiency; EGFR: Epidermal Growth Factor Receptor; EFS: Event Free Survival; IMRT: Intensity-Modulated Radiation Therapy; LAG-3: Lymphocyte-Activation Gene 3; LA-NSCLC: Locally Advanced Non-Small Cell Lung Cancer; MRD: Minimum residual disease; MSI: Microsatellite Instability; NKG2A: Natural Killer Group 2A; NSCLC: Non-Small Cell Lung Cancer; OS: Overall Survival; pCR: Pathologic Complete Response; PD1: Programmed Death 1; PD-L1: Programmed Death-Ligand 1; PFS: Progression-Free Survival; RT: Radiotherapy; SBRT: Stereotactic Body Radiation Therapy; TMB: Tumor Mutational Burden.
